# Lepidopteran Genomes Have Denser Transposable Elements in Smaller Chromosomes, Likely Driven by Non-allelic Homologous Recombination

**DOI:** 10.1093/gbe/evaf137

**Published:** 2025-07-12

**Authors:** Hyerin An, Kiwoong Nam

**Affiliations:** DGIMI, INRAE, University of Montpellier, Montpellier, France; DGIMI, INRAE, University of Montpellier, Montpellier, France

**Keywords:** transposable elements, nonallelic homologous recombination, genome evolution, chromosome size, Lepidoptera

## Abstract

Transposable elements (TEs) drive major genome size and structural variations, yet evolutionary forces affecting their accumulation and removal remain unclear. Classical models predict that higher recombination rates lead to more efficient purifying selection, such as TE removal. However, in the painted lady butterfly (*Vanessa cardui)*, smaller chromosomes harbor denser TE content than larger ones despite higher recombination rates. This unexpected pattern raises questions about whether similar trends occur across other Lepidoptera species and what evolutionary forces are behind this pattern. Across ten species spanning ten lepidopteran families, we investigated the relationship between chromosome size and TE organization using comparative genomics. We observed that smaller chromosomes consistently have higher TE densities in all the investigated species. Chromosome size had positive correlations with average inter-TE distance for both young (<5% divergence) and old TEs (5% to 10% divergence). However, the ratio of these distances (young/old TEs) was negatively correlated with chromosome size in eight of ten species, with two showing no statistically significant correlation, suggesting that smaller chromosomes have higher removal rates of sequence between TEs, potentially due to nonallelic homologous recombination, causing the loss of unique sequences between nonallelic homologs. Population genomics analyses showed inconsistent correlations between chromosome size and genetic diversity or selection coefficients between *Danaus plexippus* and *Spodoptera frugiperda*, ruling out the efficiency of purifying selection or selective constraint as the main driver. Taken together, we demonstrate that Lepidoptera has a unique genomic feature of denser TEs in smaller chromosomes, with nonallelic homologous recombination as a potential driving force.

Significance This study reveals that smaller chromosomes have denser transposable elements in Lepidoptera. While population genomics revealed variation in effective population sizes among chromosomes, no consistent signature of reduced purifying selection was detected. Instead, we propose that nonallelic homologous recombination may drive preferential sequence removal between TE insertions in smaller chromosomes, contributing to compact TE clustering over time.

## Introduction

Transposable elements (TEs) constitute a significant fraction of eukaryotic genome content, contributing to genome size variation across species (Elliott and Gregory 2015). TE insertions may disrupt functional genetic elements (McDonald et al. 1997), promote ectopic recombination between non-homologous loci (Langley et al. 1988; Montgomery et al. 1991), or interfere with gene expression (Maksakova et al. 2006). The insertion of TEs is generally harmful to their hosts (Kidwell and Lisch 1997), although instances of adaptive TE domestication have been documented (Almeida et al. 2022). Consequently, host genomes regulate TE accumulation through processes including epigenetic silencing, purifying selection, and recombination-mediated removal (Hollister and Gaut 2009; Barrón et al. 2014). The evolutionary dynamics of TEs are shaped by both insertional activity and elimination, under the influence of natural selection, recombination, and demography (Charlesworth and Langley 1989; Wright and Schoen 1999; Le Rouzic and Capy 2005; Dolgin and Charlesworth 2008; González and Petrov 2009; Blumenstiel 2011; Barrón et al. 2014; Chuong et al. 2017; Bourque et al. 2018).

The negative correlation between recombination rate and TE density has been observed from almost all Eukaryotic species (Bartolome et al. 2002; Rizzon et al. 2002; Jensen-Seaman et al. 2004; Tian et al. 2009; Daron et al. 2014; Shen et al. 2017). Low recombination rates reduce the efficacy of purifying selection, leading to a higher likelihood of TE insertions becoming fixed in these regions. The enrichment of TEs was also observed in pericentromeric and heterochromatic regions, where recombination rates are low (Dolgin and Charlesworth 2008; Kent et al. 2017; Wang et al. 2023).

However, recent evidence from Shipilina et al. (2022) reports an opposite TE distribution pattern in the painted lady butterfly (*Vanessa cardui*), a lepidopteran species. First, they showed that smaller chromosomes have higher recombination rates. This pattern is expected since each chromosomal arm necessitates at least one crossing over during meiotic cell division, irrespective of chromosome length (Pardo-Manuel de Villena and Sapienza 2001; Smith and Nambiar 2020). Indeed, the negative correlation between chromosome length and recombination rate has been reported across Eukaryotes, including insects (Lindsley et al. 1997), mammals (Nachman and Churchill 1996; Broman et al. 1998; Lander et al. 2001; Kong et al. 2002; Matise et al. 2007), plants (Gion et al. 2016; Shen et al. 2017), and birds (Rodionov 1996; International Chicken Genome Sequencing Consortium 2004; Stapley et al. 2008; Kawakami et al. 2014) (but exceptional cases also exist; Beye et al. 2006). Second, unexpectedly, Shipilina et al. (2022) also showed that smaller chromosomes display higher TE densities than larger ones. Their multiple regression analysis shows that this pattern cannot be generated by TE insertional preferences for smaller chromosomes or by lower selective constraints in these chromosomes. Hence, the evolutionary forces responsible for this pattern remain unidentified. It also remains uncertain whether this trend is specific to the painted lady or extends to higher taxonomic levels, such as Lepidoptera or higher phylogenetic ranks within Hexapoda.

Promoted by the study on the painted lady butterfly, we aim to uncover the evolutionary forces responsible for this pattern. We analyze chromosome-sized genome assemblies from ten families of the Lepidoptera order and 32 families of seven non-lepidopteran orders in Hexapoda (Coleoptera, Collembola, Diptera, Hemiptera, Hymenoptera, Neuroptera, and Thysanoptera) to test correlations between TE distribution and chromosome size. We also analyzed resequencing data of *Spodoptera frugiperda* (Noctuidae) and *Danaus plexippus* (Nymphalidae) to investigate the effect of purifying selection on the distribution of TEs.

## Results

### Chromosome-sized Assemblies

On the National Center for Biotechnology Information (NCBI), there were 2,130 genome sequences of Lepidoptera, including 316 chromosome-sized assemblies from 297 species as of the 16th of January 2023. Out of these assemblies, reference gene annotations were available for 38 assemblies across 36 species spanning ten families (Bombycidae, Crambidae, Gelechiidae, Lycaenidae, Noctuidae, Nymphalidae, Papilionidae, Plutellidae, Sphingidae, and Tortricidae). To prevent potential bias from unequal sampling numbers among families, we randomly selected one species from each of the ten families that cover seven superfamilies within the Lepidoptera (Bombycoidea, Gelechioidea, Noctuoidea, Papilionoidea, Pyraloidea, Tortricoidea, and Yponomentoidea), characterized by their high species diversity (Cranston and Gullan 2009). The assembly size of the ten lepidopteran species varied nearly 3-fold, ranging from 248.7Mb in *D. plexippus* to 783.4Mb in *Chilo suppressalis*. The chromosome number also varied among the species, ranging from 23 in *Aricia agestis* to 31 in *Plutella xylostella* and *S. frugiperda*. The completeness of the ten genome assemblies was determined using Benchmarking Using Single-Copy Orthologs (BUSCO) (Manni et al. 2021). The proportions of complete BUSCO genes consistently exceeded 96%, except for *Leguminivora glycinivorella* (93.0%; Table 1). The N50 values of the assemblies ranged from 9.2Mb to 28.3Mb (mean N50 = 19Mb).

**Table 1:** Genome assemblies used in this study

Species	Family	Assembly	Accession	Genome size (Mb)	Chromosome number	Scaffold N50 (Mb)	BUSCO score
*Aricia agestis*	Lycaenidae	ilAriAges1.1	GCF_905147365.1	435.3	23	19	97.4%
*Bombyx mori*	Bombycidae	Bmori_2016v1.0	GCF_014905235.1	460.3	28	16.8	98.7%
*Chilo suppressalis*	Crambidae	PGI_CHILSU_V6	GCA_902850365.2	783.4	30	28.3	98.2%
*Danaus plexippus*	Nymphalidae	Dplex_v4	GCA_009731565.1	248.7	30	9.2	98.3%
*Iphiclides podalirius*	Papilionidae	IP_504.v2_0	GCA_933534255.1	430.7	30	15.1	96.6%
*Leguminivora glycinivorella*	Tortricidae	LegGlyc_1.1	GCF_023078275.1	657.4	28	25.3	93.0%
*Manduca sexta*	Sphingidae	JHU_Msex_v1.0	GCF_014839805.1	470	28	14.2	98.3%
*Pectinophora gossypiella*	Gelechiidae	ilPecGoss1.1	GCF_024362695.1	476.5	30	16.7	98.7%
*Plutella xylostella*	Plutellidae	ilPluXylo3.1	GCF_932276165.1	323.3	31	11.3	98.0%
*Spodoptera frugiperda*	Noctuidae	ver6.0	GCA_019297735.1	384.5	31	13.2	97.5%

Species names, families, assembly names in NCBI genomes, and assembly accession numbers are listed. In addition, genome size, chromosome number, scaffold N50, and the BUSCO score (the proportion of complete BUSCO genes) are also shown for each assembly.

### Higher Repeat Densities on Smaller Chromosomes Only in Lepidoptera

**Fig. 1.**
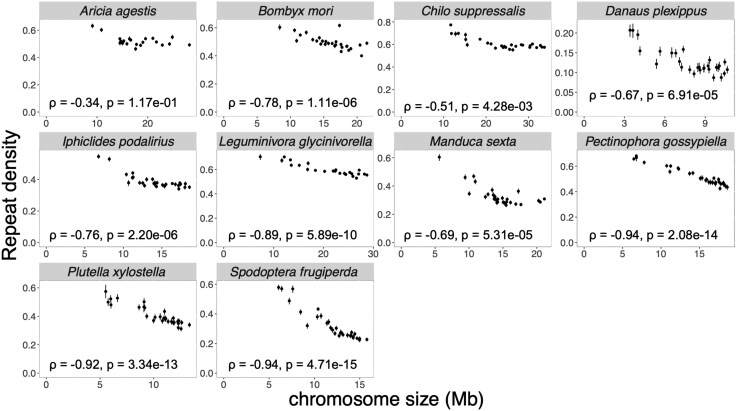
The relationship between chromosome length and the density of repeats in Lepidoptera. Each dot represents the density of repeats in each chromosome, with error bars indicating 95% bootstrapping confidence intervals calculated from 100-kb windows with 1,000 replications. Spearman's correlation coefficients (*ρ*) and the corresponding FDR-corrected *P*-values are shown.

Repeats constituted on average 42% of the genome, ranging from 11.9% in *D. plexippus* to 59.6% in *Chilo suppressalis*. TEs accounted for approximately 75% to 97% of the total repeats, representing 31.5% to 40.7% of the genome (Fig. S1). These TEs included Long Interspersed Nuclear Elements (LINEs, from 41.7% in *Aricia agestis* to 74.6% in *Bombyx mori*), Long Terminal Repeats (LTRs, from 1.9% in *D. plexippus* to 23.5% in *Pectionophora gossypiella*), Short Interspersed Nuclear Elements (SINEs, from 1.9% in *Chilo suppressalis* to 21.3% in *S. frugiperda)*, and DNA transposons (from 10.9% in *S. frugiperda* to 24.6% in *Chilo suppressalis*). First, we calculated the proportion of repeats for each chromosome (Fig. 1). All investigated lepidopteran families consistently showed negative correlations between chromosome lengths and repeat densities (Spearman's correlation coefficients ρ ranged between −0.34 and −0.94). These negative correlations were statistically significant from all families (FDR-adjusted *P*-value < 0.05), except for *A. agestis* (ρ = −0.34, FDR-adjusted *P*-value = 0.117). This result indicates that the previously reported negative correlation between chromosome lengths and repeat densities in the painted lady butterfly (Shipilina et al. 2022) was observed across all examined lepidopteran families.

We used multiple linear regression to test if the observed pattern in Fig. 1 is a byproduct of a correlation with the potential differential sequence context of insertional preference or selection constraints against TE insertion using GC content and coding sequence (CDS) densities. Statistically significant negative correlations between chromosome lengths and repeat densities persisted even after accounting for the effects of GC content and CDS densities (*P*-value < 0.05) in all families except Bombycidae (Bombyx mori, *t*-value = −1.1, *P*-value = 0.291) (Table S2). For example, in *S. frugiperda* (Table S3), the repeat density was negatively correlated with the chromosome size after controlling for GC content and CDS density (*t*-value = −10.15, *P*-value = 2.37 × 10^−10^). The multiple regression model also revealed negative correlations between CDS and repeat densities from four out of ten families. The GC content showed a statistically significant correlation (*P*-value < 0.05) with repeat density in four families: positive in three, negative in one. This result means that a consistent pattern of GC content or CDS density across all families was not observed, unlike repeat density.

Correlation tests were also conducted for each of the major families of TE to test whether a specific TE family drove the observed negative correlations. We focused on five TE families, including DNA transposons, LINEs, LTRs, rolling circles, and SINEs. Chromosome size showed negative correlations with repeat densities in 42 out of 50 cases (10 families × 5 TE types), with 36 of these correlations being statistically significant (FDR-adjusted *P*-value < 0.05). Specifically, the density of LINE, which is the most abundant family of TEs, had negative correlations with chromosome sizes in all ten families with statistical significance (FDR-adjusted *P*-value < 0.05), except for *A. agestis* (*ρ* = 0.25, FDR-adjusted *P*-value = 0.270) (Fig. S2). We also computed correlations for the five most prevalent sub-families of LINEs to test whether specific LINE sub-families were responsible for generating negative correlations. The correlations between repeat proportion and chromosome size were negative with statistical significance (FDR-adjusted *P*-value < 0.05) in almost all LINE sub-families (48 out of 50) (Fig. S3a).

**Fig. 2.**
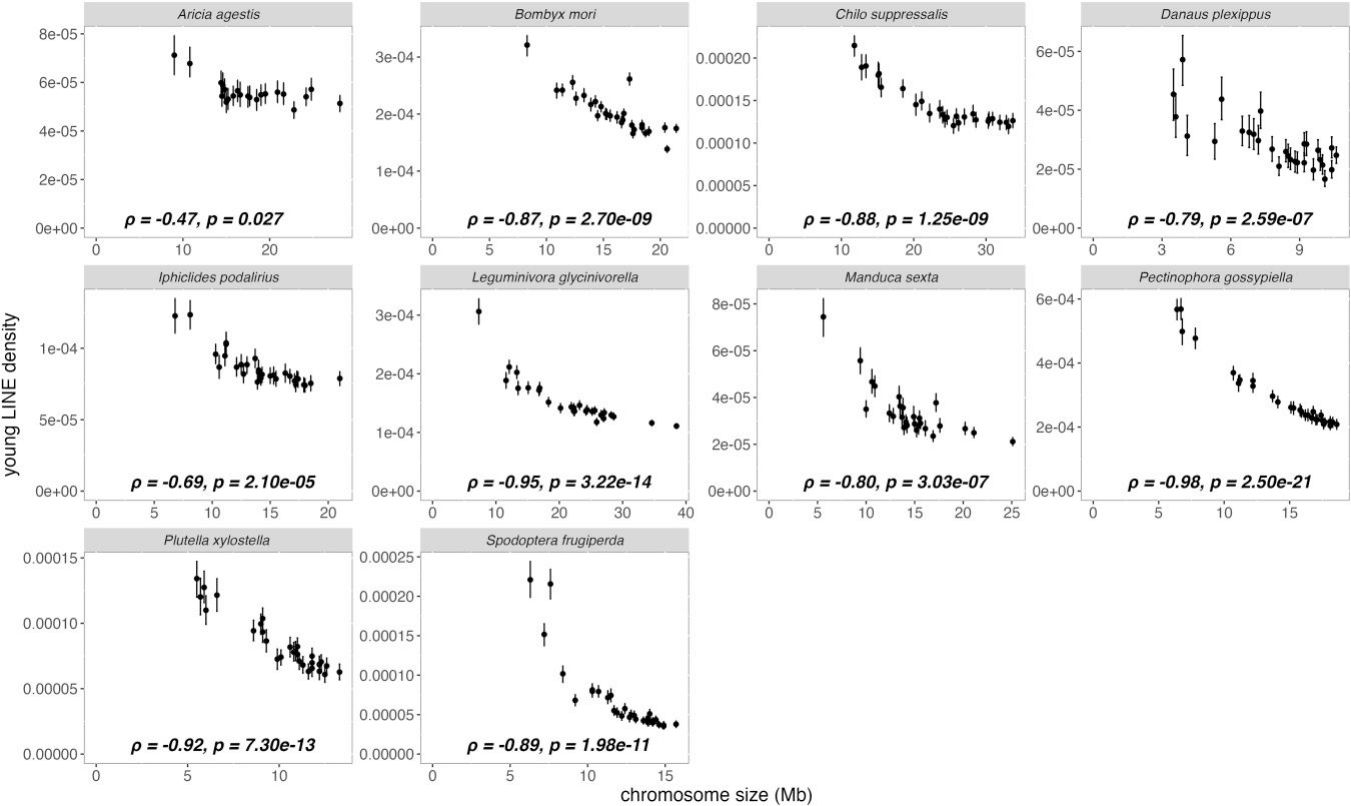
Smaller chromosomes have a higher proportion of young LINEs across all ten lepidopteran species. The plots depict the relationship between chromosome length and the density of young LINEs, of which the divergence from master sequences is less than 0.05. Error bars represent 95% bootstrapping confidence intervals calculated from 100-kb windows with 1,000 replications. Spearman's correlation coefficients (*ρ*) and corresponding *P*-values are shown for each plot.

The observed correlations might stem from an anecdotal evolutionary event preceding the split into the lineages leading to the extant lepidopteran families. To test this possibility, we conducted a correlation test on young LINEs, of which divergence to their master TE sequences was less than 5% (see Fig. S4 for distribution of divergence). The negative correlations between chromosome sizes and young TE densities were observed for all ten families with statistical significance (FDR-adjusted *P*-value < 0.05) (Fig. 2), implying that an anecdotal evolutionary event is not likely to cause the observed correlations. The negative correlations were also observed across all SINE sub-families (Fig. S3b) and young SINEs (Fig. S5).

**Fig. 3.**
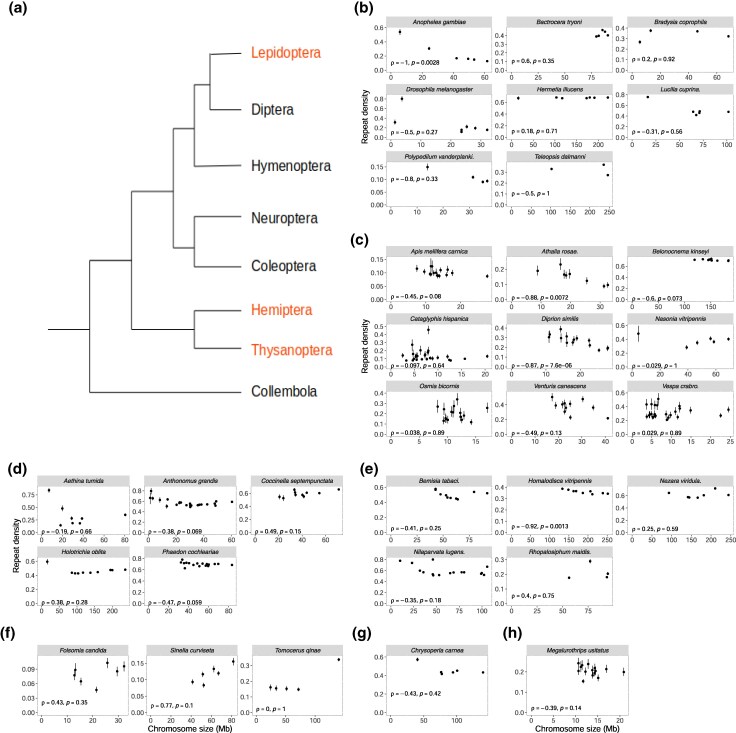
No consistent correlational pattern in non-lepidopteran orders. a) Topology of hexapod orders analyzed in our study (Wheeler et al. 2001). The orders in red are holocentric orders. The plot illustrates the relationship between chromosomal lengths and the proportion of repeats in seven other hexapod orders: b) Diptera, c) Hymenoptera, d) Coleoptera, e) Hemiptera, f) Collembola, g) Neuroptera, and h) Thysanoptera. Error bars indicate 95% bootstrapping confidence intervals calculated from 100-kb windows with 1,000 replications. Spearman's correlation coefficients (*ρ*) and corresponding *P*-values are shown for each plot.

We tested the correlation between chromosome length and repeat density across non-lepidopteran 32 families within seven hexapod orders, including Coleoptera, Collembola, Diptera, Hemiptera, Hymenoptera, Neuroptera, and Thysanoptera (listed in Table S1). Hemiptera and Thysanoptera are holocentromeric orders, like Lepidoptera, and the rest are monocentromeric. Among the investigated families, 11 from five orders exhibited positive correlations, while 21 from six orders displayed negative correlations (Fig. 3). None of these orders demonstrated consistent correlation trends. Even when focusing on species with chromosome lengths comparable to those of Lepidoptera, such as Hymenoptera and Thysanoptera, no consistent pattern was observed in the relationship between chromosome size and TE density (Figs. 3c and h). Since repetitive DNA elements in eukaryotes are frequently linked with heterochromatin (Spence et al. 1998; Henikoff et al. 2001; Mandrioli et al. 2003; Hill et al. 2009; Malik and Henikoff 2009), we analyzed the *S. frugiperda* genome to test whether the higher repeat density in smaller chromosomes could be attributed to telomeric repeats. However, our analysis did not reveal a significantly elevated density of TEs in the telomeric regions (Fig. S6), as observed in *Melitaea cinxia*, another lepidopteran species (Ahola et al. 2014).

### Increased Sequence Loss Rate in Smaller Chromosomes

**Fig. 4.**
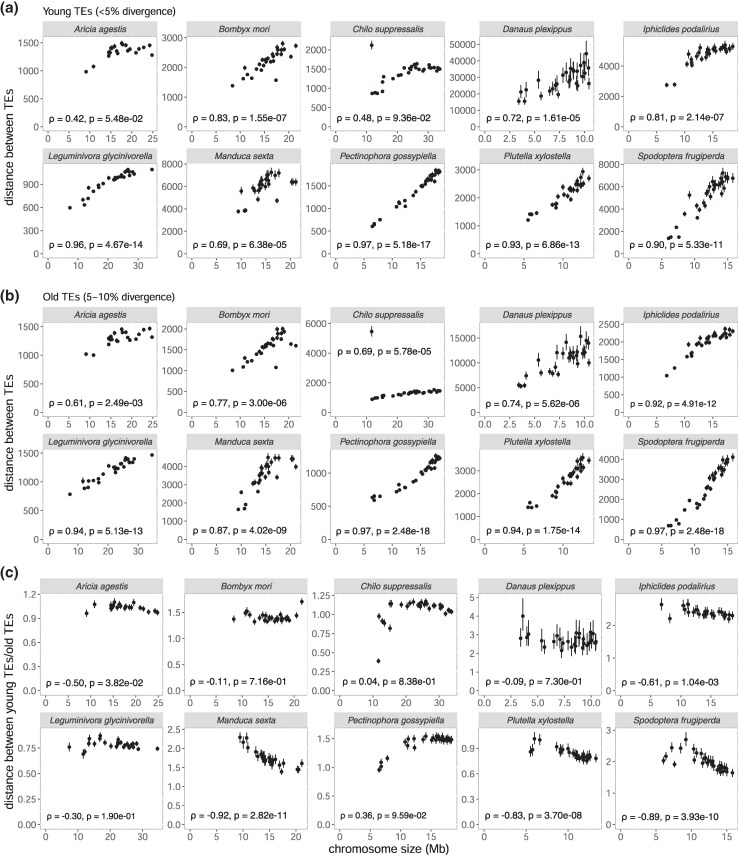
Relationship between chromosome size and inter-TE distances by TE age category. The plots show the correlation between chromosome size and a) the average inter-TE distance for young TE insertions (divergence <5%) b) the average inter-TE distance for old TE insertions (divergence between 5% and 10%) c) the ratio of average distances between young and old TEs (<5%/5% to 10% divergence). Each point represents a chromosome from one of ten lepidopteran species. In most species, TE insertions are more tightly clustered in smaller chromosomes, especially for old TEs, suggesting a role for sequence removal over time. Error bars indicate 95% bootstrapping confidence intervals calculated from 100-kb windows with 1,000 replications. Spearman's correlation coefficients (*ρ*) and corresponding *P*-values for significant analyses are shown for each plot.

To further explore potential links between chromosome structure and TE dynamics, we measured the average distance between adjacent TEs on each chromosome. We classified TEs according to their sequence divergence from the consensus: young TEs (< 5% divergence) and old TEs (5% to 10% divergence). In all species studied, the average distance between young TEs showed a positive correlation with chromosome size (*ρ* = 0.42 to 0.97) (Fig. 4a), with statistical significance in eight of the ten species (FDR-adjusted *P* < 0.05). In *Chilo suppressalis* and *Aricia agestis*, the correlations remained positive but were not statistically significant. For old TEs, all ten species showed a statistically significant positive correlation with chromosome size (*ρ* = 0.61 to 0.97, FDR-adjusted *P* < 0.05). This result suggests that shorter chromosomes tend to have shorter distances between TEs (Figs. 1 and 2), which is expected since these chromosomes have denser TEs (Fig. 2).

If newly inserted TEs increase the distance between existing TEs, smaller chromosomes with higher TE insertion rates are expected to have a longer distance between existing TEs than larger chromosomes. However, the ratio of the distance between young TEs to old TEs was negatively correlated with chromosome size in eight out of ten species (*ρ* between −0.09 and −0.92) (Fig. 4c), which is the opposite pattern from the expectation. In the two remaining species, no significant correlation was detected. This pattern implies that smaller chromosomes have an increased rate of sequence removal over evolutionary time.

### Inconsistent Evidence of Reduced Selection in Smaller Chromosomes

The negative correlations between repeat densities and chromosome lengths can arise if smaller chromosomes have less efficient purifying selection to remove polymorphic TE insertions in a population than larger ones. To test this possibility, we analyzed two resequencing datasets from two species. The first dataset is a publicly available one of *S. frugiperda* (Gimenez et al. 2020) generated from 32 individuals in the USA. We excluded the Z chromosome because the sex chromosome often exhibits unique evolutionary patterns and differences in genetic content compared to autosomes. Indeed, in *S. frugiperda*, significant selective sweeps have been reported on the Z chromosome (Durand et al. 2022; Fiteni et al. 2022). The total number of analyzed autosomal single-nucleotide polymorphisms (SNPs) was 15,862,530. The second dataset was generated from *D. plexippus* (Nymphalidae) using publicly available whole-genome resequencing data (Zhan et al. 2014) from 74 samples across three geographic regions: the Caribbean and Central/South America, the Pacific areas, and Iberian and North African areas. After excluding the Z chromosome, a total of 19,184,866 SNPs remained.

**Fig. 5.**
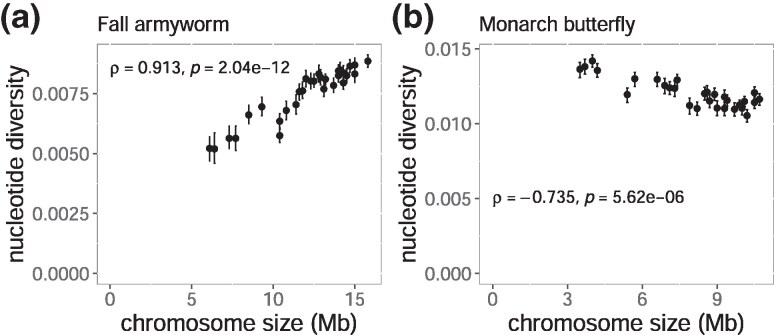
Relationship between chromosome size and nucleotide diversity in *S. frugiperda* and *D. plexippus* has a smaller effective population size. The plots show the relationship between chromosome size and nucleotide diversity calculated using pixy. Error bars indicate 95% bootstrapping confidence intervals calculated from 100-kb windows with 1,000 replications. Spearman's correlation coefficients (*ρ*) and corresponding *P*-values are shown for each plot.

**Fig. 6.**
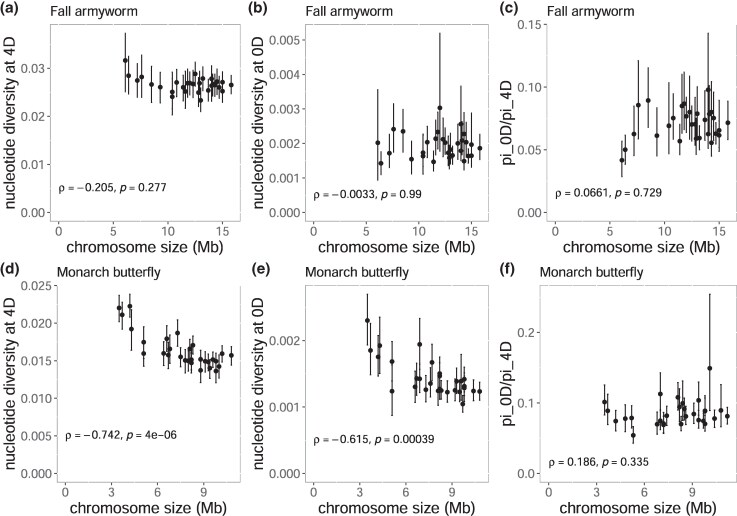
Relationship between chromosome size and nucleotide diversity at degenerate coding sites. The plots show the correlation between chromosome size and nucleotide diversity at (a, d) 4-fold degenerate sites, (b, e) 0-fold degenerate sites, and (c, f) the 0D/4D *π* ratio, as a proxy for the relative efficiency of purifying selection, for each chromosome in *Spodoptera frugiperda* (a–c) and *Danaus plexippus* (d–f). Error bars indicate 95% bootstrapping confidence intervals calculated from 100-kb windows with 1,000 replications. Spearman's correlation coefficients (*ρ*) and corresponding *P*-values for significant analyses are shown for each plot.

We first compared nucleotide diversity (*π*) among chromosomes as a proxy for effective population size based on the rationale that purifying selection efficiency is a function of local effective population size (Corbett-Detig et al. 2015). In *S. frugiperda*, chromosome size showed a significant positive correlation with *π* (*ρ* = 0.91, *P*-value = 2.04 × 10^−12^, Fig. 5a and Fig. S7), ranging between 5.19 × 10^−3^ and 8.85 × 10^−3^. On the other hand, in *D. plexippus*, *π* had a negative correlation with chromosome size (*ρ* = 0.73, *P*-value = 5.62 × 10^−6^, Fig. 5b), ranging between 1.05 × 10^−2^ and 1.42 × 10^−2^. In *D. plexippus*, *π* at 4-fold degenerate sites (4D) showed a strong and statistically significant negative correlation with chromosome size (Fig. 6d, *ρ* = −0.74, *P*-value = 4.00 × 10⁻⁶). In *S. frugiperda,* the correlation was also negative but not significant (Fig. 6a, *ρ* = −0.21, *P*-value = 0.277). These patterns are consistent with the previously reported ones in other lepidopteran species (Martin et al. 2016; Cicconardi et al. 2021; Mackintosh et al. 2022). Concerning diversity at 0-fold degenerate sites (0D), *D. plexippus* showed a significant correlation (Fig. 6e, *ρ* = −0.61, *P*-value *=* 3.90 × 10^−4^), unlike *S. frugiperda* (Fig. 6b, *ρ* = −0.0033, *P*-value *=* 0.990). As a result, the ratio of 0D to 4D diversity, which can be used as a proxy for efficiency of purifying selection, displayed statistically non-significant trends in *S. frugiperda* (Fig. 6c, *ρ* = 0.066, *P*-value *=* 0.729) and in *D. plexippus* (Fig. 6f, *ρ* = 0.19, *P*-value *=* 0.335). Together, these results do not support the association between chromosome size and the efficacy of purifying selection.

**Fig. 7.**
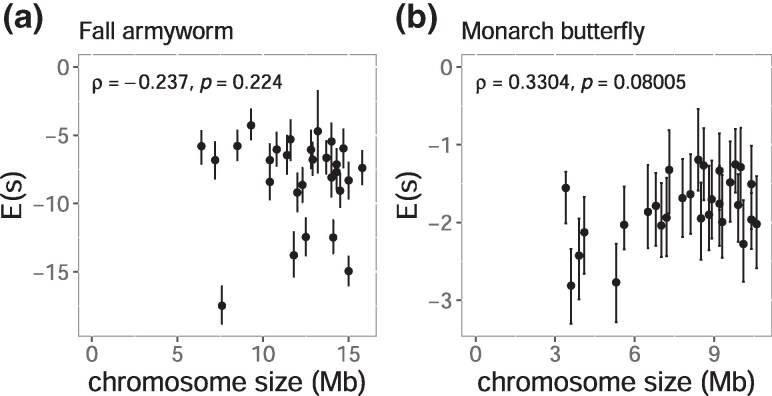
Correlations between chromosome size and the average selection coefficient of deleterious mutations (**Es**). Error bars indicate 95% bootstrapping confidence intervals calculated from 100-kb windows with 1,000 replications. Spearman's correlation coefficients (*ρ*) and corresponding *P*-values are shown for each plot.

We estimated the chromosome average selection coefficient of deleterious mutations using the allele frequency spectrum of nonsynonymous and synonymous SNVs. Selection coefficients of deleterious mutations revealed no consistent correlation with chromosome size between *S. frugiperda* (*ρ* = −0.24, *P*-value = 0.224) and *D. plexippus* (*ρ* = 0.33, *P*-value = 0.0801) (Fig. 7), not supporting the explanation that the observed TE pattern was generated by a differential level of selective constraint among the chromosomes.

## Discussion

To understand the evolutionary forces shaping genome architecture, we investigated the relationship between chromosome size and TE across ten lepidopteran families. The correlations between chromosome sizes and repeat densities were negative across all ten families (Fig. 1) across various types of repeats (Fig. S2), consistent with the report of Wright et al. (2024) who independently observed the same trend in Lepidoptera. This negative correlation was observed even after controlling for the effect of GC content and CDS densities on repeat densities. This pattern was consistently observed irrespective of different ages and subtypes of LINEs (Fig. 2, Fig. S3), the most prevalent TE families in lepidopteran genomes. This trend was observed exclusively from Lepidoptera among the eight studied orders (Figs. 1 and 3). A spatial distribution of repeats (Fig. S6) shows that the observed correlation was not due to the telomeric effect (Meyne et al. 1989; Daron et al. 2014; Wang et al. 2023). We found that in all ten investigated lepidopteran species, the average distance between TEs increases with chromosome size, both for young (< 5% divergence) and old (5% to 10% divergence) TE insertions, as expected from the pattern of denser TEs in smaller chromosomes. To explore how the spacing between TEs changes over time, we calculated the ratio of the average distance between young TEs to the one between old TEs for each chromosome. Unexpectedly, this ratio was negatively correlated with chromosome size in eight out of ten species, meaning that smaller chromosomes have relatively shorter distances between old TEs compared to young TEs than larger chromosomes, despite higher insertion rates of young TEs on smaller chromosomes. This pattern can be explained by higher removal rates of sequences between TEs in smaller chromosomes over evolutionary time.

We propose nonallelic homologous recombination (NAHR) as a potential mechanism of the denser TEs in smaller chromosomes of Lepidoptera. NAHR is a recombination-based process between homologous but nonallelic sequences located at different genomic positions, often leading to structural variations such as deletions, duplications, and translocations (Hoang et al. 2010; Startek et al. 2015). Because NAHR requires sequence homology, it is frequently mediated by repetitive elements such as transposable elements, especially when they are abundant and closely spaced. The frequency of NAHR is typically positively correlated with the recombination rate (Inoue and Lupski 2002; Lindsay et al. 2006; Turner et al. 2008; Völker et al. 2010; Liu et al. 2012). Turner et al. (2008) directly measured NAHR events in human sperm. They found deletion bias of NAHR, often at a 2:1 ratio. Consistent with this trend, genome-wide analyses have shown that NAHR is responsible for extensive deletions over evolutionary time (Petrov et al. 2003; Hedges and Deininger 2007; Kent et al. 2017), removing up to nearly 1 Mb of sequence from the human genome within a few million years (Sen et al. 2006; Han et al. 2007; Cordaux 2008). Thus, we suggest that smaller chromosomes with higher recombination rates experience more NAHR-mediated loss of unique sequences between nonallelic homologs, causing denser TE densities than larger ones.

As an alternative mechanism, we analyzed resequencing data from *S. frugiperda* and *D. plexippus* to test whether weaker purifying selection targeting TE insertion among chromosomes generated higher TE density. The direction of the correlation between *π* and chromosome size differed. In *S. frugiperda*, smaller chromosomes exhibited lower diversity, whereas in *D. plexippus*, smaller chromosomes displayed higher diversity, not exhibiting a general correlative pattern between chromosome size and local effective population size. The efficiency of purifying selection was also tested using the 0D/4D *π* ratio, assuming that *π* at 0D and 4D sites reflects genetic diversities under incomplete purifying selection and neutral evolution, respectively. In both *S. frugiperda* and *D. plexippus*, the trend was positive, but neither correlation was statistically significant. These results again suggest that differences in purifying selection efficiency are unlikely to explain the consistent accumulation of TEs in smaller chromosomes. Smaller chromosomes do not have lower selection coefficients of spontaneous deleterious mutations than larger chromosomes, also suggesting that genetic elements on smaller chromosomes are unlikely to be selectively less constrained.

Another possibility is that smaller chromosomes may have lower recombination rates than larger ones in the investigated lepidopteran species. In lepidopteran species with available recombination maps, smaller chromosomes consistently exhibit higher recombination rates than larger ones (Fig. S8). In addition, the wood white butterfly revealed a negative correlation between chromosome size and recombination rate, although with relatively weak statistical support for this observation (*ρ* = −0.292, *P*-value = 0.06) (Palahí I Torres et al. 2023). The authors suggest that the higher *P*-value mainly stems from an unusually elevated recombination rate in a single chromosome rather than a general trend across all chromosomes. Consequently, it is unlikely that most lepidopteran species deviate from the typical Eukaryotic pattern, making this scenario less plausible.

## Conclusion

In this study, we observed across all ten analyzed lepidopteran families that smaller chromosomes consistently have higher TE densities than larger ones, a pattern not observed in other hexapod orders. This result is unexpected under classical population genetic theory, which predicts more efficient purging of deleterious insertions in smaller chromosomes due to their higher recombination rates. We propose NAHR as a plausible explanation of this pattern. In Lepidoptera, the higher recombination rate in smaller chromosomes may increase the likelihood of NAHR events between homologous TEs or other repetitive elements. This process can lead to the preferential deletion of inter-TE sequences, including unique sequences, ultimately resulting in the progressive clustering of TEs over evolutionary time. Future research should aim to characterize the molecular mechanisms and genetic contexts of NAHR to uncover the evolutionary forces driving the unique genomic features of Lepidoptera.

## Materials and Methods

### Chromosomal Genome Assemblies

As of January 16, 2023, the NCBI database contained 2,130 genome sequences of Lepidoptera, including 316 chromosome-sized assemblies from 297 species. Reference gene annotations were available for 38 assemblies across 36 species within ten families (Bombycidae, Crambidae, Gelechiidae, Lycaenidae, Noctuidae, Nymphalidae, Papilionidae, Plutellidae, Sphingidae, and Tortricidae). To avoid potential bias from unequal sampling among families, we randomly selected one species from each of the ten families, which represent seven superfamilies (Bombycoidea, Gelechioidea, Noctuoidea, Papilionoidea, Pyraloidea, Tortricoidea, and Yponomeutoidea) known for their high species diversity (Cranston and Gullan 2009), followed by downloading the reference genomes and gene annotations from these ten species. Accession numbers, genome sizes, chromosome numbers, and taxonomic information for all genomes are given in Table S1. We also downloaded chromosome-level reference genome assemblies with gene annotation from 32 non-lepidopteran families in Hexapoda, including five coleopteran, three collembolan, eight dipteran, nine hymenopteran, one neuropteran, five hemipteran, and one thysanopteran genomes. Again, one species per family has been chosen randomly.

The accuracy of the ten lepidopteran genome assemblies was evaluated using the Benchmarking Universal Single-Copy Orthologs (BUSCO) analysis with the lepidoptera_odb10 database in busco-5.2.2 (Manni et al. 2021).

### Genomic Feature Distributions Between Chromosomes

The libraries of TE master sequences were generated for each species using RepeatModeler2 v2.0.3 (Flynn et al. 2020). Repetitive elements were identified and classified using RepeatMasker (version 4.1.0) (https://www.repeatmasker.org/) on each genome using the classification of TE master sequences from these libraries, followed by the calculation of repeat densities in 100 kb windows with custom Python scripts (see Script Availability Statement). The ages of TE were calculated from the proportion of sequence difference from the library sequences. The GC content and CDS density in 100-kb windows were calculated from the FASTA files for the reference genome assemblies and from the GTF files for the gene annotation, respectively. We calculated the average repeat density, GC content, and CDS density in each chromosome as well as 95% bootstrapping confidence intervals calculated by 1,000 resampling replications on 100-kb windows. Spearman's correlation test was performed and multiple-testing correction involving false-discovery rate using the R package stats (v.4.5.0, R Core Team 2024).

### Calculation of Inter-TE Distances

To investigate the spatial distribution of TEs across chromosomes, we analyzed RepeatMasker output files to compute the average distances between TE insertions at two divergence intervals: <5% and between 5% and 10%, assuming that the divergence reflects the age of TE insertions. The pairwise distances between adjacent TEs belonging to the same divergence category were calculated, followed by calculating the mean distances for each category for each chromosome and the ratio of these two.

### Population Genomics Analyses on S. Frugiperda and D. Plexippus

For *S. frugiperda*, we used the SNP dataset generated from 32 corn-strain individuals from the USA (Gimenez et al. 2020) for the population genomics analysis. In this study, adaptor sequences from the fastq files were removed using AdapterRemoval-2.1.7 (Schubert et al. 2016), and mapping against the reference genome (NCBI Genome Accession number: GCA_019297735.1) was performed using Bowtie2-2.3.4.1 (Langmead and Salzberg 2012). Variants were called using GATK-v4.0.11.0 (McKenna et al. 2010) with HaplotypeCaller, followed by discarding SNPs with low-quality scores.

The gene annotation was performed using maker (Cantarel et al. 2008), available on the BioInformatics Platform for Agroecosystem Arthropods (https://bipaa.genouest.org/sp/spodoptera_frugiperda_pub/download/annotation/corn/OGS6.1/). VCF annotations to nonsynonymous or synonymous SNPs were generated by snpEff 5.2c (Cingolani et al. 2012). Detailed information is available in Gimenez et al. (2020). The total number of analyzed autosomal SNPs was 15,862,530.

For *D. plexippus*, we downloaded fastq files from 80 samples across three geographic regions, including populations from South Florida (around Miami), the Caribbean, and Central/South America, from the Pacific (including Oceania), and from across the Atlantic (spanning Iberia and North Africa [Zhan et al. 2014]; NCBI SRA Project ID: PRJNA258112). Fastq filtering, mapping, variant calling, and filtering were performed using the same methods with the reference genome assembly from NCBI (DUEA00000000), except for the version of the GATK (v4.1.2.0). We excluded six samples with abundant missing genotypes, reducing the sample size from 80 to 74. Finally, 9,184,866 SNPs remained for the analyses. VCF annotations to nonsynonymous or synonymous SNPs were generated by snpEff 5.2c (Cingolani et al. 2012).

### Calculation of Nucleotide Diversity Across All Sites, 4D and 0D Coding Sites, and 0D/4D Ratios

To assess the variation in purifying selection efficiency across chromosomes, we calculated *π* for three categories: all sites, 4D sites, and 0D sites. With the assumption that *π* of 0D sites is affected by incomplete purifying selection while that of 4D sites reflects the level of neutral genetic diversity, the 0D/4D *π* ratio was used as a proxy for the relative efficacy of purifying selection across chromosomes. Coding site types were annotated using the codingSiteTypes.py script from the *genomics_general* toolkit (https://github.com/simonhmartin/genomics_general), which classifies coding positions based on codon degeneracy from CDS and GFF files. We generated BED files corresponding to 0D and 4D sites from the output. We generated an all-site VCF to retain both variant and invariant positions. Variant quality was filtered with the same criteria as the abovementioned variant filtering. Low-quality SNPs were recorded in a BED file and excluded from the all-site VCF using VCFtools v0.1.16 (Danecek et al. 2011) with the –exclude-bed and –recode options. We calculated *π* using pixy v1.2.11 (Korunes and Samuk 2021). For 4D and 0D sites, we subset the filtered all-site VCF using the respective BED files and run pixy with a window size of 100 kb. For all-site *π*, we ran pixy directly on the filtered all-site VCF, computing chromosome-wide *π* values across all genomic positions. For each chromosome, *π* values were averaged across all windows to obtain a mean estimate per site class. The 0D/4D *π* ratio was computed by dividing the average *π* at 0D sites by the corresponding value at 4D sites.

### Calculating the Distribution of Fitness Effect

The distribution of fitness effects of new deleterious mutations was estimated using within-species nucleotide polymorphism data with DFE-alpha-release-2.16 (Keightley and Eyre-Walker 2007; Eyre-Walker and Keightley 2009). For this estimation, we first calculated the folded site frequency spectrum from synonymous and nonsynonymous SNPs, which were used to reflect neutral evolution and the potential for deleterious mutations, respectively. Then, DFE-alpha calculated the DFE using the maximum likelihood framework.

## Supplementary Material

evaf137_Supplementary_Data

## Data Availability

This study is based on publicly available data of whole-genome assemblies (please see Table S1) and resequencing datasets (NCBI SRA Project ID: PRJNA494340, PRJNA577869, PRJNA258112). The vcf file used for population genomics analyses is available at Zenodo for *S. frugiperda* (https://zenodo.org/records/4024047) and *D. plexippus* (https://zenodo.org/records/15322345). The computer programming script used to generate this vcf file is also available at Zenodo (https://zenodo.org/records/4024357). All computer programming scripts are available on GitHub (https://github.com/hye-rin-an/TE_NAHR).
