# Supplementary material for: Lepidopteran Genomes Have Denser Transposable Elements in Smaller Chromosomes, Likely Driven by Non-allelic Homologous Recombination
Source: Genome Biol Evol. 2025 Jul 12;17(8):evaf137. doi: 10.1093/gbe/evaf137 (PMC12308830; doi:10.1093/gbe/evaf137)
Supplement: evaf137_Supplementary_Data [file evaf137_supplementary_data.docx]

**Supporting Information for**

Lepidopteran genomes have less efficient purifying selection with denser transposable elements in smaller chromosomes

Hyerin An, Kiwoong Nam*

DGIMI, INRAE, Univ Montpellier, Montpellier, France.

*Author for Correspondence: Kiwoong Nam, DGIMI, INRAE, Montpellier, Fax: +33 (0)4 67 14 42 99, email address: ki-woong.nam@inrae.fr

**This Supporting Information includes.**

Tables S1 to S3

Figures S1 to S8

**Table S1. Genome assemblies information**. The cable contains the assembly names, species, NCBI accession numbers, genome sizes, chromosome numbers, and taxonomic information.

| **Category** | **Order** | **Family** | **Species** | **Assembly Name** | **Accession** | **Genome Size (Mb)** | **Chromosome Number** |
| --- | --- | --- | --- | --- | --- | --- | --- |
| **Holocentric** | Lepidoptera | Lycaenidae | *Aricia agestis* | ilAriAges1.1 | GCF_905147365.1 | 435.3 | 23 |
|  |  | Bombycidae | *Bombyx mori* | Bmori_2016v1.0 | GCF_014905235.1 | 460.3 | 28 |
|  |  | Crambidae | *Chilo suppressalis* | PGI_CHILSU_V6 | GCA_902850365.2 | 783.4 | 30 |
|  |  | Nymphalidae | *Danaus plexippus* | Dplex_v4 | GCA_009731565.1 | 248.7 | 30 |
|  |  | Papilionidae | *Iphiclides podalirius* | IP_504.v2_0 | GCA_933534255.1 | 430.7 | 30 |
|  |  | Tortricidae | *Leguminivora glycinivorella* | LegGlyc_1.1 | GCF_023078275.1 | 657.4 | 28 |
|  |  | Sphingidae | *Manduca sexta* | JHU_Msex_v1.0 | GCF_014839805.1 | 470 | 28 |
|  |  | Gelechiidae | *Pectinophora gossypiella* | ilPecGoss1.1 | GCF_024362695.1 | 476.5 | 30 |
|  |  | Plutellidae | *Plutella xylostella* | ilPluXylo3.1 | GCF_932276165.1 | 323.3 | 31 |
|  |  | Noctuidae | *Spodoptera frugiperda* | ver6.0 | GCA_019297735.1 | 384.5 | 31 |
|  | Hemiptera | Aleyrodidae | *Bemisia tabaci* | PGI_BMITA_v3 | GCA_918797505.1 | 609.9 | 10 |
|  |  | Cicadellidae | *Homalodisca vitripennis* | UT_GWSS_2.1 | GCF_021130785.1 | 2300 | 9 |
|  |  | Pentatomidae | *Nezara viridula* | PGI_NEZAVIv3 | GCA_928085145.1 | 1200 | 7 |
|  |  | Delphacidae | *Nilaparvata lugens* | ASM1435652v1 | GCF_014356525.1 | 1100 | 16 |
|  |  | Aphididae | *Rhopalosiphum maidis* | ASM367621v3 | GCF_003676215.2 | 326 | 4 |
|  | Thysanoptera | Thripidae | *Megalurothrips usitatus* | Mus_1.0 | GCA_026979955.1 | 238.1 | 16 |
| **Monocentric** | Diptera | Culicidae | *Anopheles gambiae* | AgamP3 | GCF_000005575.2 | 265 | 6 |
|  |  | Tephritidae | *Bactrocera tryoni* | CSIRO_BtryS06_freeze2 | GCF_016617805.1 | 570.6 | 5 |
|  |  | Sciaridae | *Bradysia coprophila* | BU_Bcop_v1 | GCF_014529535.1 | 309.7 | 4 |
|  |  | Drosophilidae | *Drosophila melanogaster* | Release 6 plus ISO1 MT | GCF_000001215.4 | 143.7 | 7 |
|  |  | Stratiomyidae | *Hermetia illucens* | iHerIll2.2.curated.20191125 | GCF_905115235.1 | 1000 | 7 |
|  |  | Calliphoridae | *Lucilia cuprina* | ASM2204524v1 | GCF_022045245.1 | 409.2 | 6 |
|  |  | Chironomidae | *Polypedilum vanderplanki* | Pv_5.2 | GCA_018290095.1 | 119 | 4 |
|  |  | Diopsidae | *Teleopsis dalmanni* | ASM223713v2 | GCF_002237135.1 | 623.7 | 3 |
|  | Coleoptera | Nitidulidae | *Aethina tumida* | icAetTumi1.1 | GCF_024364675.1 | 260 | 8 |
|  |  | Curculionidae | *Anthonomus grandis* | icAntGran1.3 | GCF_022605725.1 | 697.5 | 24 |
|  |  | Coccinellidae | *Coccinella septempunctata* | icCocSept1.1 | GCF_907165205.1 | 398.8 | 10 |
|  |  | Scarabaeidae | *Holotrichia oblita* | ASM2369052v1 | GCA_023690525.1 | 1400 | 10 |
|  |  | Chrysomelidae | *Phaedon cochleariae* | PGI_PHAECO_v4 | GCA_918026855.4 | 869.7 | 17 |
|  | Neuroptera | Chrysopidae | *Chrysoperla carnea* | inChrCarn1.1 | GCF_905475395.1 | 560.2 | 6 |
|  | Hymenoptera | Apidae | *Apis mellifera carnica* | ASM1384124v2 | GCA_013841245.2 | 226 | 16 |
|  |  | Athaliidae | *Athalia rosae* | iyAthRosa1.1 | GCF_917208135.1 | 172 | 8 |
|  |  | Cynipidae | *Belonocnema kinseyi* | B_treatae_v1 | GCF_010883055.1 | 1500 | 10 |
|  |  | Formicidae | *Cataglyphis hispanica* | ULB_Chis1_1.0 | GCF_021464435.1 | 206.4 | 26 |
|  |  | Diprionidae | *Diprion similis* | iyDipSimi1.1 | GCF_021155765.1 | 270.2 | 14 |
|  |  | Pteromalidae | *Nasonia vitripennis* | Nvit_psr_1.1 | GCF_009193385.2 | 297.3 | 6 |
|  |  | Megachilidae | *Osmia bicornis* | iOsmBic2.1 | GCF_907164935.1 | 223 | 16 |
|  |  | Ichneumonidae | *Venturia canescens* | ASM1945775v1 | GCF_019457755.1 | 290.8 | 11 |
|  |  | Vespidae | *Vespa crabro* | iyVesCrab1.2 | GCF_910589235.1 | 229.3 | 25 |
|  | Collembola | Sminthuridae | *Allacma fusca* | qeAllFusc8.1 | GCA_947179485.1 | 392.8 | 6 |
|  |  | Entomobryidae | *Sinella curviseta* | ASM411504v3 | GCA_004115045.3 | 364.3 | 6 |
|  |  | Tomoceridae | *Tomocerus qinae* | ASM2005564v1 | GCA_020055645.1 | 334.4 | 5 |

**Table S2.** Summary of the multiple linear regression analysis with proportion of total repeats as the dependent variable, and chromosome length, GC content, and coding sequence density as explanatory variables in lepidopteran species. ***, **, and * indicates statistical significance with p < 0.001, p < 0.01, and p < 0.05, respectively.

| **Species** | **Variable** | **Estimate** | **Standard error** | **t-value** | **p-value** | **R^2^** | **DF** | **Global**  **p-value** | **Shapiro-Wilk**  **p-value** |
| --- | --- | --- | --- | --- | --- | --- | --- | --- | --- |
| ***Aricia agestis*** | **Intercept** | 0.24510 | 0.33315 | 0.736 | 0.47139 | 0.3567 | 18 | 0.01177 | 0.897 |
|  | **GC content** | 0.39241 | 0.26168 | 1.500 | 0.15106 |  |  |  |  |
|  | **CDS density** | -0.15472 | 0.09786 | -1.581 | 0.13128 |  |  |  |  |
|  | **Chromosome size** | -0.08517 | 0.02620 | -3.251 | 0.00443 ** |  |  |  |  |
| ***Bombyx mori*** | **Intercept** | 0.16635 | 0.14573 | 1.141 | 0.26494 | 0.8275 | 24 | 6.287 × 10^-10^ | 0.188 |
|  | **GC content** | 0.51630 | 0.06804 | 7.589 | 7.93 × 10^-8^ *** |  |  |  |  |
|  | **CDS density** | -0.11488 | 0.03318 | -3.463 | 0.00202 ** |  |  |  |  |
|  | **Chromosome size** | -0.02753 | 0.02550 | -1.079 | 0.29118 |  |  |  |  |
| ***Chilo suppressalis*** | **Intercept** | 0.202933 | 0.114208 | 1.777 | 0.0812 | 0.6221 | 54 | 4.342 × 10^-12^ | 0.091 |
|  | **GC content** | 0.462761 | 0.092765 | 4.989 | 6.67 × 10^-6^ *** |  |  |  |  |
|  | **CDS density** | -0.149337 | 0.029389 | -5.081 | 4.80 × 10^-6^ *** |  |  |  |  |
|  | **Chromosome size** | -0.053859 | 0.009198 | -5.855 | 2.91 × 10^-7^ *** |  |  |  |  |
| ***Danaus plexippus*** | **Intercept** | 0.389930 | 0.043031 | 9.062 | 2.25 × 10^-9^ *** | 0.7835 | 25 | 4.452 × 10^-9^ | 0.857 |
|  | **GC content** | -0.129052 | 0.047114 | -2.739 | 0.0112 * |  |  |  |  |
|  | **CDS density** | 0.025355 | 0.020453 | 1.240 | 0.2266 |  |  |  |  |
|  | **Chromosome size** | -0.060686 | 0.006172 | -9.832 | 4.51 × 10^-10^ *** |  |  |  |  |
| ***Leguminivora glycinivorella*** | **Intercept** | 0.923910 | 0.183398 | 5.038 | 4.25 × 10^-5^ *** | 0.8332 | 23 | 1.021 × 10^-9^ | 0.369 |
|  | **GC content** | 0.037657 | 0.114438 | 0.329 | 0.745 |  |  |  |  |
|  | **CDS density** | -0.008202 | 0.044122 | -0.186 | 0.854 |  |  |  |  |
|  | **Chromosome size** | -0.120916 | 0.011744 | -10.296 | 4.39 × 10^-10^ *** |  |  |  |  |
| ***Mandua sexta*** | **Intercept** | 0.32088 | 0.29959 | 1.071 | 0.2948 | 0.7354 | 24 | 9.758 × 10^-8^ | 0.900 |
|  | **GC content** | 0.94421 | 0.31101 | 3.036 | 0.0057 ** |  |  |  |  |
|  | **CDS density** | -0.08530 | 0.06779 | -1.258 | 0.2204 |  |  |  |  |
|  | **Chromosome size** | -0.11812 | 0.04476 | -2.639 | 0.0144 * |  |  |  |  |
| ***Pectionophora gossypiella*** | **Intercept** | 0.74546 | 0.13464 | 5.537 | 9.36 × 10^-6^ *** | 0.9524 | 25 | < 2.2× 10^-16^ | 0.406 |
|  | **GC content** | 0.09740 | 0.07978 | 1.221 | 0.23356 |  |  |  |  |
|  | **CDS density** | -0.10883 | 0.03192 | -3.409 | 0.00222 ** |  |  |  |  |
|  | **Chromosome size** | -0.21282 | 0.01327 | -16.037 | 1.15 × 10^-14^ *** |  |  |  |  |
| ***Plutella xylostella*** | **Intercept** | 0.75914 | 0.19731 | 3.847 | 0.000695 *** | 0.8784 | 26 | 1.224 × 10^-12^ | 0.643 |
|  | **GC content** | -0.02988 | 0.12761 | -0.234 | 0.816715 |  |  |  |  |
|  | **CDS density** | -0.08560 | 0.05903 | -1.450 | 0.159031 |  |  |  |  |
|  | **Chromosome size** | -0.24139 | 0.01727 | -13.979 | 1.33 × 10^-13^ *** |  |  |  |  |
| ***Iphiclides podalirius*** | **Intercept** | 0.79075 | 0.23801 | 3.322 | 0.00275 ** | 0.7514 | 25 | 2.463 × 10^-8^ | 0.716 |
|  | **GC content** | 0.08778 | 0.11776 | 0.745 | 0.46296 |  |  |  |  |
|  | **CDS density** | 0.01939 | 0.04478 | 0.433 | 0.66881 |  |  |  |  |
|  | **Chromosome size** | -0.13773 | 0.04111 | -3.350 | 0.00257 ** |  |  |  |  |

**Table S3: Multiple linear regression test in *Spodoptera frugiperda*.** The correlation between repeat density and chromosome length was tested when the effect of GC content and CDS density in repeat density was controlled.

|  | Estimate | Standard Error | t-value | p-value |
| --- | --- | --- | --- | --- |
| (Intercept) | 0.136157 | 0.034241 | 3.976 | 5.26 ✕10^-4^ |
| GC content | 0.041627 | 0.022186 | 1.876 | 7.23 ✕10^-2^ |
| CDS density | -0.015217 | 0.006714 | -2.267 | 3.23✕10^-2^ |
| chromosome size | -0.061134 | 0.006023 | -10.150 | 2.37✕10^-10^ |

Residual standard error: 0.01454 on 25 degrees of freedom

Multiple R-squared: 0.8191, Adjusted R-squared: 0.7974

F-statistic: 37.74 on 3 and 25 DF, p-value: 1.954 ✕ 10^-9^

Shapiro-Wilk normality test on the residuals: W = 0.98076, p-value = 0.857

**Figure S1. Bar plots showing the proportion (%) of different repeated elements in the genomes of lepidopteran species.** The most abundant components were LINEs, LTR, DNA elements, and SINEs in most species (except in *Danaus plexippus*)**Figure S2**. **Spearman’s correlation test between TE density and chromosome size for each class of TE.** Each bar represents Spearman's correlation coefficients (ρ), and nonsignificant correlations (FDR-corrected p-values > 0.05) are colored with hatched lines.

**Figure S3A**. **Smaller chromosomes have a higher proportion of all sub-families of LINEs**. Relationship between the chromosome length and proportion of the most abundant LINE sub-families. Each color represents a sub-family of LINE. The error bars indicate 95% bootstrap confidence intervals calculated from 100kb windows with 1,000 replications.

**Figure S3B**. **Smaller chromosomes have a higher proportion of all sub-families of SINEs**. Relationship between the chromosome length and proportion of the most abundant SINE sub-families. Each color represents a sub-family of SINE. The error bars indicate 95% bootstrap confidence intervals calculated from 100kb windows with 1,000 replications.

**Figure S4.**
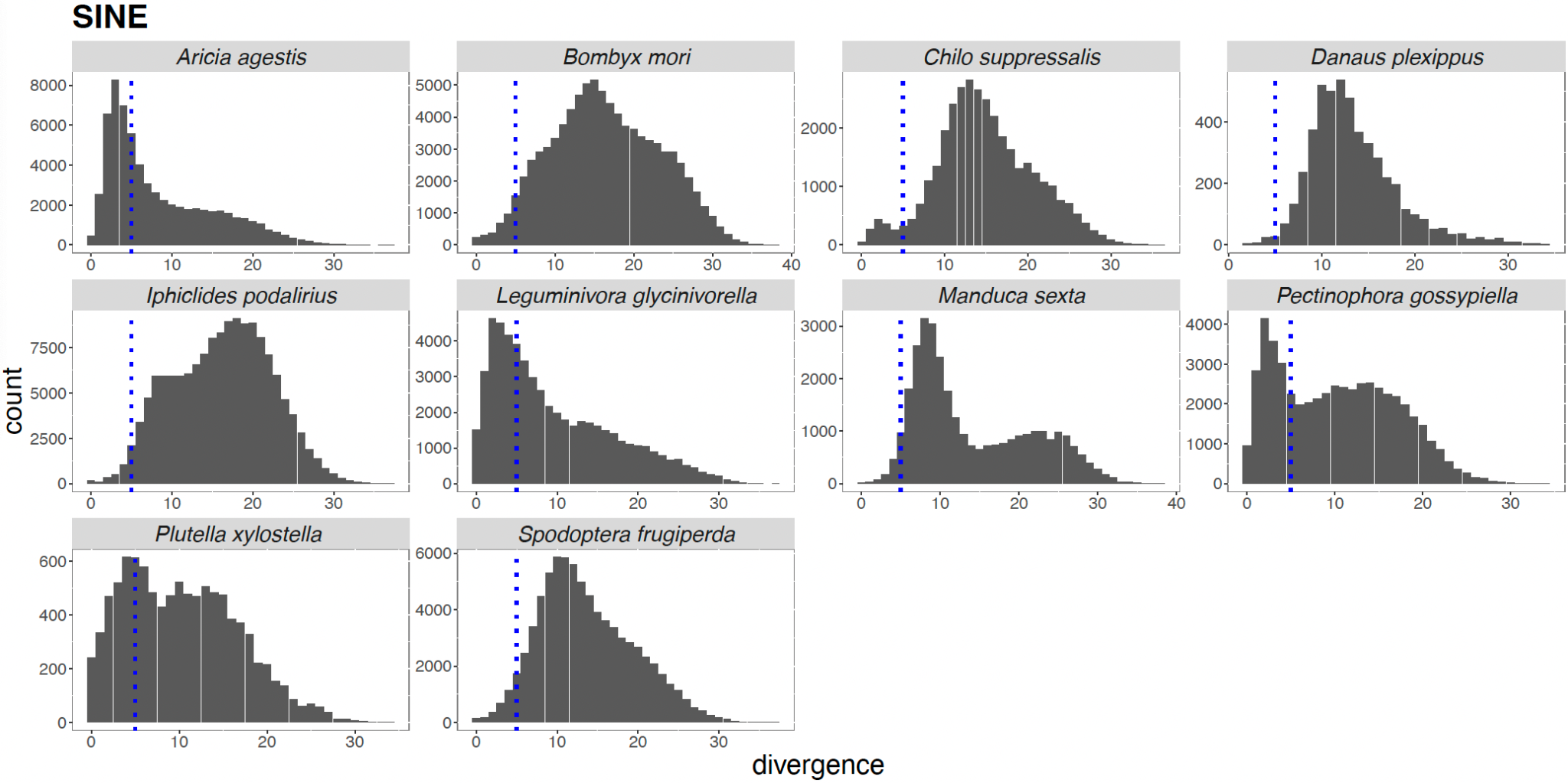

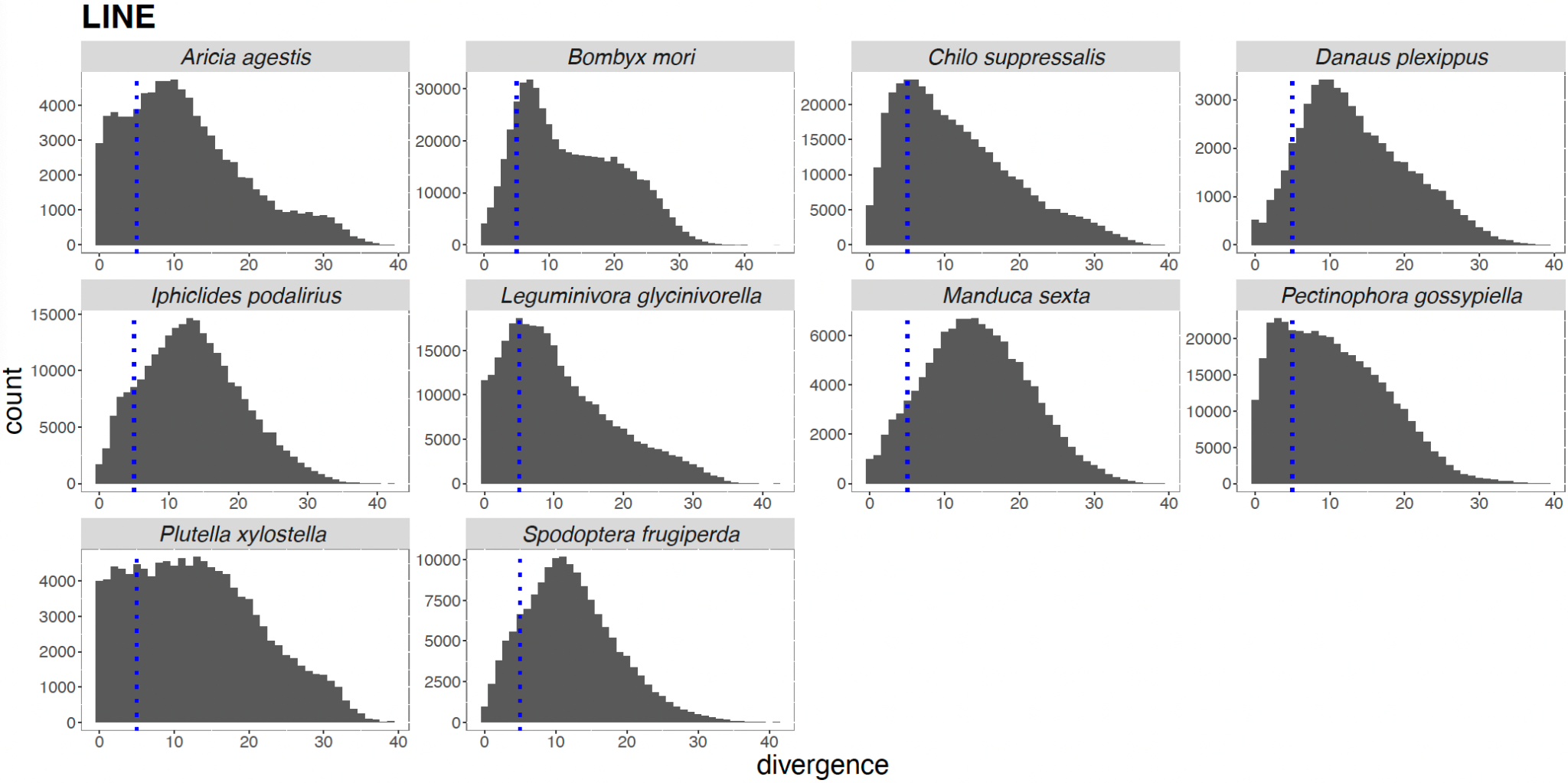
Distribution of the percentage of divergence of LINE and SINE from the master sequence.


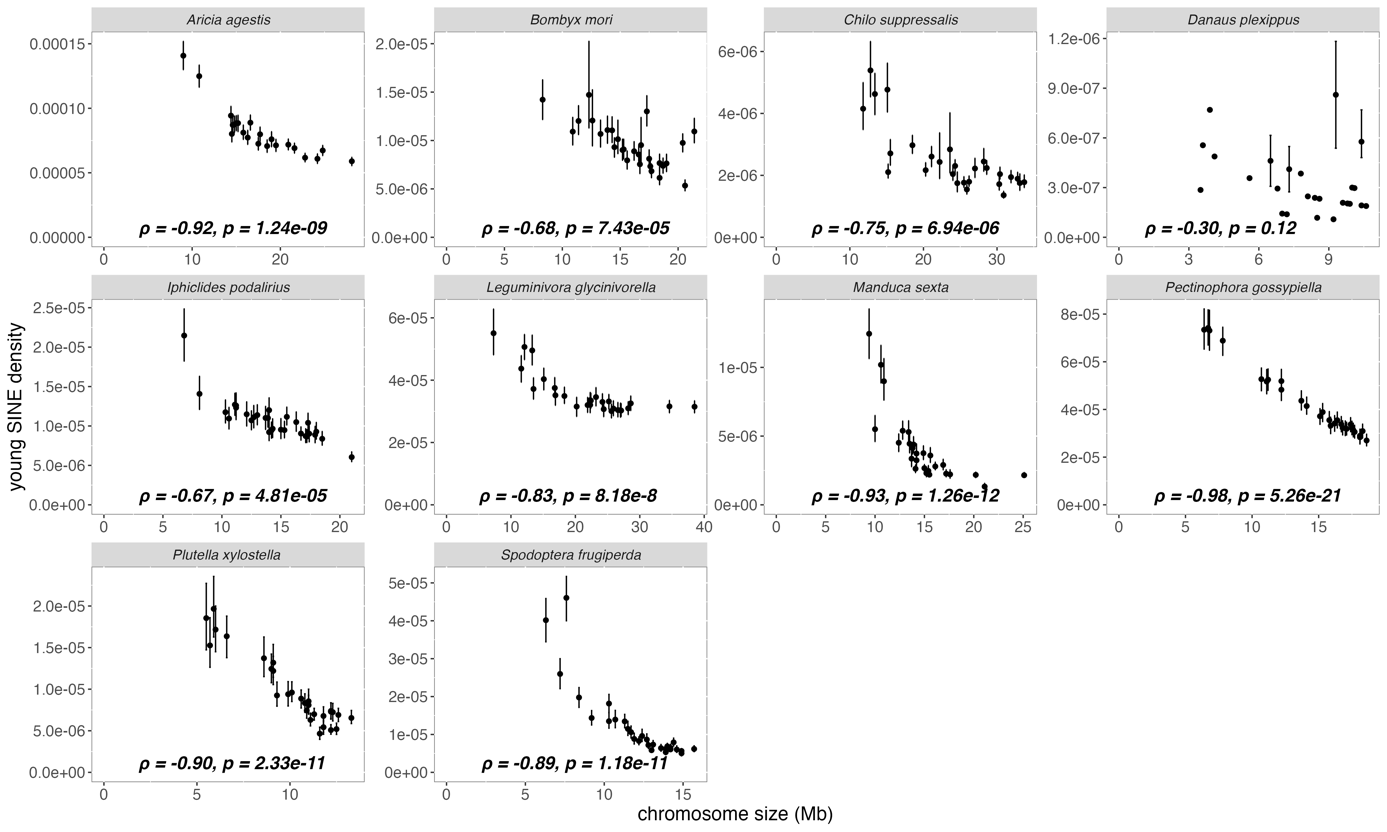


**Figure S5. Smaller chromosomes have a higher proportion of young SINEs across all ten lepidopteran species.** The plots depict the relationship between chromosome length and the density of young SINEs, of which the divergence from master sequences is less than 0.05. Error bars represent 95% bootstrapping confidence intervals calculated from 100 kb windows with 1,000 replications. Spearman’s correlation coefficients (ρ) and corresponding p-values are shown for each plot.


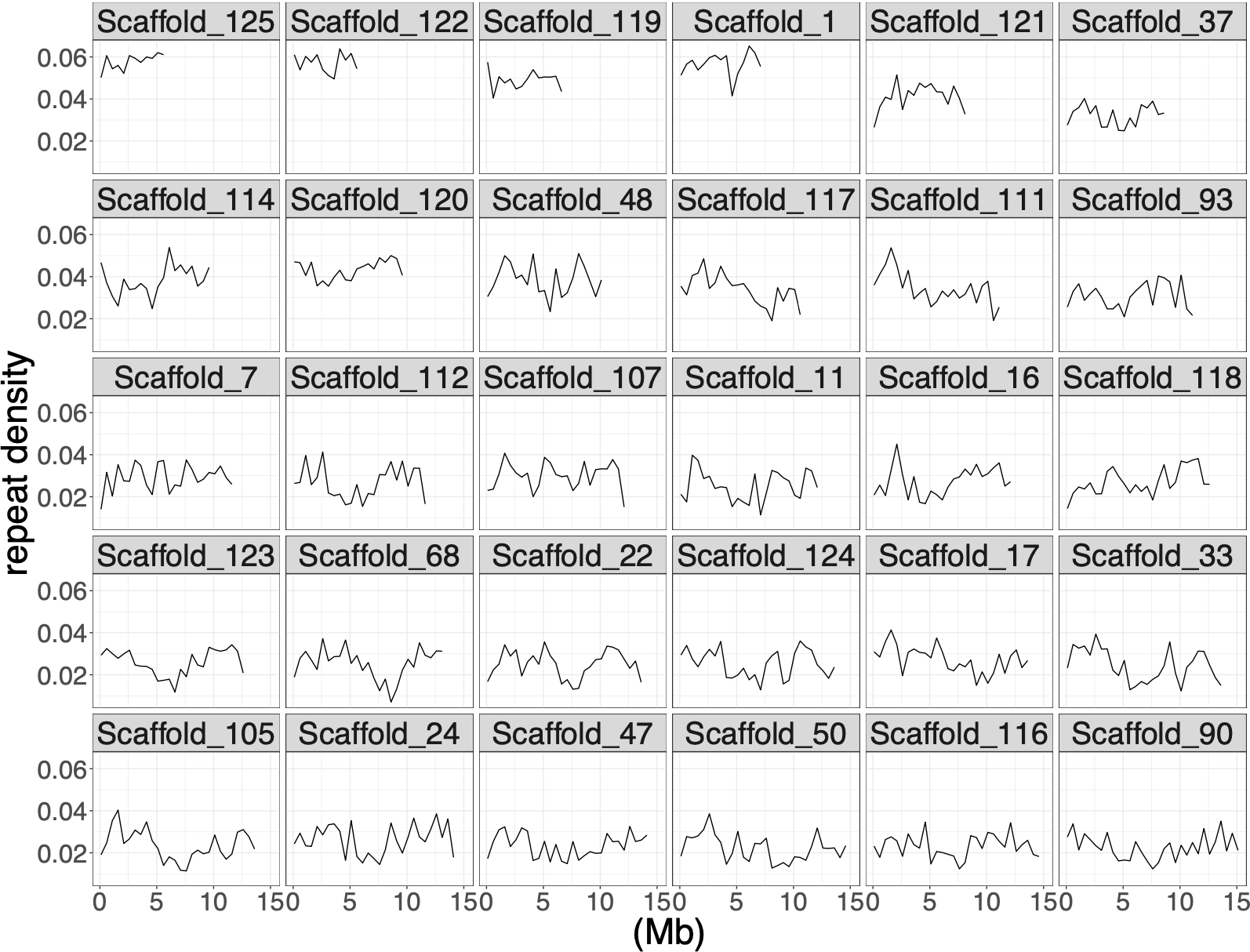


**Figure S6**. Spatial distribution of repeat density along the chromosomes of *Spodoptera frugiperda.*


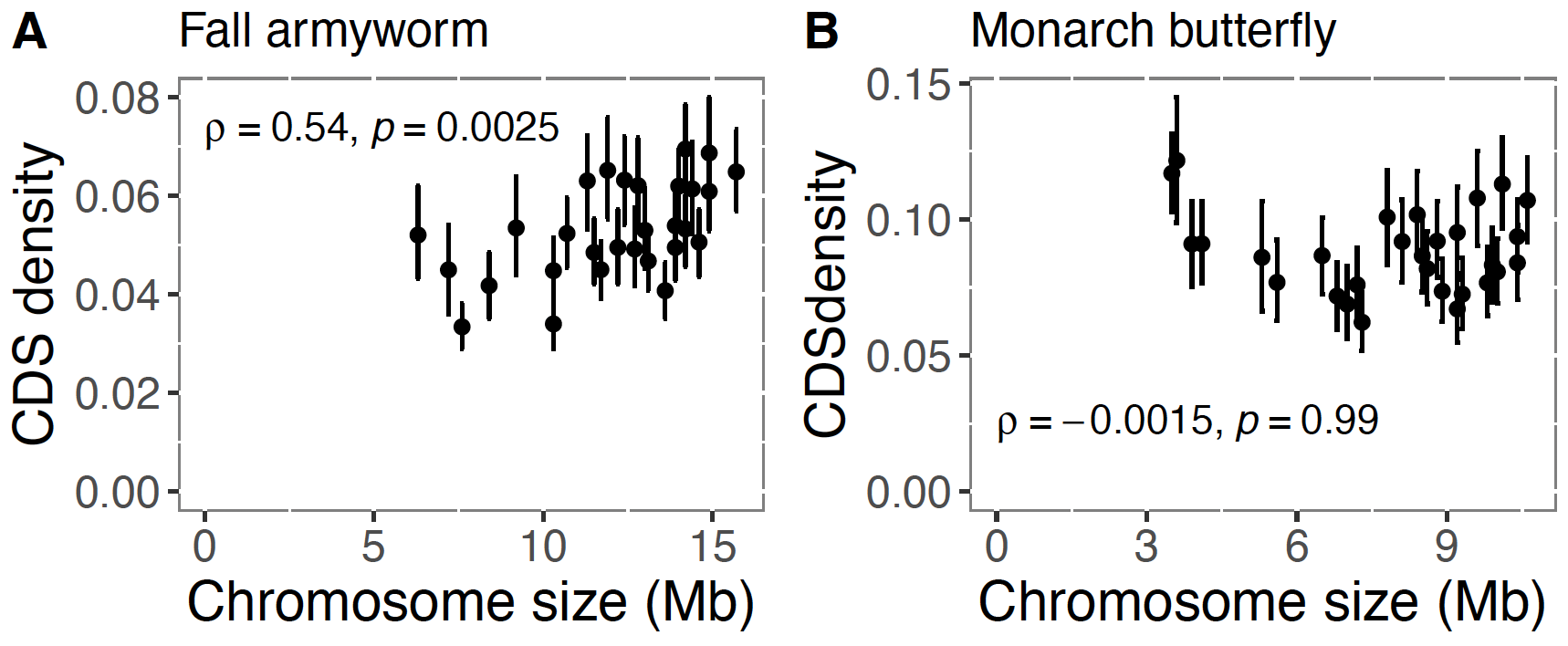


**Figure S7.** In *S. frugiperda*, the CDS density is positively correlated with chromosome sizes, but in *D. plexippus,* CDS density is not significantly correlated with chromosome sizes. Error bars indicate 95% bootstrapping confidence intervals calculated from 100 kb windows with 1,000 replications. Spearman’s correlation coefficients (ρ) and corresponding p-values are shown for each plot.

**Figure S8. The correlation between chromosome size and recombination rate in Lepidoptera.** The plots were generated using linkage maps originally presented for *Bombyx mori*
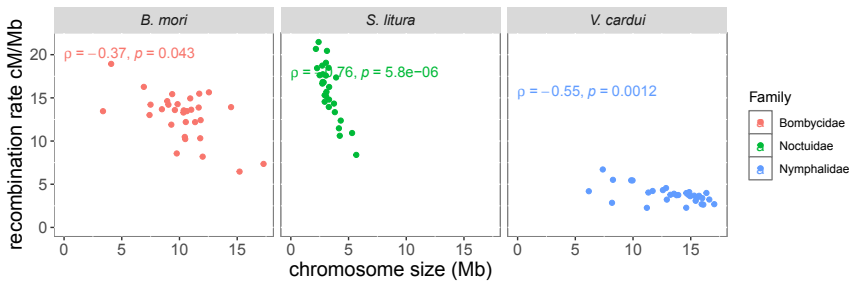
(Yamamoto et al. 2008), *Spodoptera litura* (Cheng et al. 2017), and *Vanessa cardui* (Shipilina et al. 2022).

**Reference**

Cheng T et al. 2017. Genomic adaptation to polyphagy and insecticides in a major East Asian noctuid pest. Nat. Ecol. Evol. 1:1747–1756. doi: 10.1038/s41559-017-0314-4.

Shipilina D et al. 2022. Linkage mapping and genome annotation give novel insights into gene family expansions and regional recombination rate variation in the painted lady (Vanessa cardui) butterfly. Genomics. 114:110481. https://www.sciencedirect.com/science/article/pii/S0888754322002269 (Accessed August 13, 2024).

Yamamoto K et al. 2008. A BAC-based integrated linkage map of the silkworm *Bombyx mori*. Genome Biol. 9:R21. doi: 10.1186/gb-2008-9-1-r21.
